# Overview of Biliary Diseases

**DOI:** 10.1155/1995/42965

**Published:** 1995

**Authors:** Ricardo L. Rossi


*HPB Surgery*, 1995, Vol. 9, pp. 1–4
Reprints available directly from the publisher
Photocopying permitted by license only

© 1995 OPA (Overseas Publishers Association)
Amsterdam B.V. Published in The Netherlands
by Harwood Academic Publishers GmbH
Printed in Malaysia

### Overview of Biliary Diseases*

RICARDO L. ROSSI

### GALLSTONE DISEASE

The introduction and rapid establishment of laparoscopic cholecystectomy as the standard of care in patients with symptomatic gallstones represents one of the most striking stories in biliary surgery. Studies presented at this Congress confirm that the procedure is associated with a lesser endocrine stress response; patients require less narcotic use; have a lower incidence of pulmonary complications; have a decreased hospital stay; return to work earlier; the cost per procedure can be lower than that for an open cholecystectomy; and the patients have a similar long-term improvement of quality of life as compared to the open operation. However, from a socioeconomic point of view, such savings to society have been outweighed by a substantial increase in the cholecystectomy rates (28% in one of the series), primarily patient driven, rather than secondary to liberalization of medical indications.In addition, there has been an increased incidence of retained common duct stones, as many surgeons have abandoned the use of routine intra-operative cholangiograms. This has resulted in the increased use of intravenous cholangiogram (26% in one series),increased use of ERCP (43%); and endoscopic papillotomy (250%) with a net increase of associated endoscopic complications such as pancreatitis. Bile duct injuries have also increased by 2.4 fold with a cost estimated in the United States for the treatment of the initial injury of $30,000 dollars per patient. The related medical cost in the life of the patient is estimated to be $300,000. There is an additional cost to society for the patient's disability.

With increased experience, the use of laparoscopic cholecystectomy has expanded and most contraindications are currently relative (i.e. acute cholecystitis, pregnancy, previous laparotomies etc.). A prospective study confirmed that gallbladder perforation and spillage occurs in about 1/3 of the patients, but the long-term effects can be prevented by abdominal lavage and removal of the stones. A study has confirmed that delayed surgery for acute cholecystitis appears to have no advantage over early operation. The thickness of the gallbladder wall on ultrasound has been reported as the best predictor of the surgical difficulty during laparoscopic dissection.

A major controversy is the management of common duct stones in the era of laparoscopic cholecystectomy. While the use of laparoscopic cholecystectomy expanded throughout the world like a brush fire, the use of laparoscopic common duct exploration via the cystic duct or via the common duct has increased slowly and remains the domain of a few. Although the concept of a one stage approach to the gallbladder and the common duct laparoscopically has merit, realistically its routine use appears more distant and less likely in view of the lower number of cases, the need of increased skills, the existence of alternative modalities of therapy (ERCP; open operation) and the referral patterns with gastroenterologists often dealing with the common duct stone prior to referring the patient to surgery.

Indication for preoperative endoscopic retrograde cholangiogram and the use of routine or selective intraoperative cholangiogram also remains controversial. Some continue to proclaim the merits of routine intraoperative cholangiogram to identify stones, define the anatomy and identify tumors. If so, cholangiography with fluoroscopy and imaging intensification appears to be superior, minimize operative time, minimize the number of false positive studies and therefore the number of unnecessary common duct explorations. A study evaluating the selective use of preoperative endoscopic retrograde cholangiogram and the selected use of intraoperative cholangiogram based on the estimated risk of having C.D. stones as a function of the preoperative liver functions studies, the size of the common duct on ultrasound and the previous history of pancreatitis or jaundice, reported that only 1.6% of the patients developed symptomatic common duct stones during the

*Based on papers presented at 1994 Inagural World Congress of the International Hepto-Pancreato-Biliary Association, Boston, May 31– June 3, 1994.

follow-up period. It appears that no definitive algorithm can be instituted for the management of common duct stones without considering the expertise and resources available in an institution. Although it is likely that a one stage laparoscopic approach will be gradually implemented in an increasing number of centers, the merits of other alternatives such as an open operation or endoscopic papillotomy are likely to remain. Surgeons and endoscopists can work as team to try to optimize the management of common duct stones; i.e., a surgeon with limited skills who finds an unexpected common duct stone can increase the success rate and safety of endoscopic instrumentation by placing a catheter in the cystic duct. The use of simultaneous intra-operative laparoscopic cholecystectomy and endoscopic retrograde papillotomy has been reported. However, it appears to be logistically and technically demanding.

Other diagnostic methods for common duct stones were reported such as laparoscopic ultrasound and routine cholangioscopy. However, they are expensive, require additional training and appear less likely to be used routinely.

Bile duct leaks from the cystic duct are more common following laparoscopic cholecystectomy and are treated effectively by endoscopic stenting and percutaneous drainage of a collection if needed. In one study with limited endoscopic resources, bile leaks were dealt with by prophylactic routine short term use of a sub-hepatic drain. An example of how resources affect therapeutic conduct.

Several studies reported on the merits of percutaneous cholecystostomy in the management of acute calculus and acalculus cholecystitis, particularly in high risk patients. In one study, 1 out of 26 and in another, 1 out of 17 patients required open cholecystectomy for gangrenous cholecystitis.

Reports from China and Korea attempted to shed light on the pathogenesis of primary hepatic and intra-hepatic stones, a disease prevalent in the orient. One of these excellent studies reviewed the principles in the management of these patients that include the clearance of stones, the treatment of strictures, treatment of infections and achieving adequate biliary drainage. The procedures need to be tailored to the findings on ultrasound, CT scan, cholangiography and cholangioscopy. Operations include common duct exploration over a T-tube, common duct exploration and intrahepatic exploration with a high hepaticojejunostomy, hepatic resections and combination of such procedures. Recurrences are frequent.

Continued challenges in the management of gallstone disease are: to better define the role of laparoscopic and endoscopic procedures in the management of common duct stones; to decrease the incidence of introgenic injuries, and to lower cost of procedures by rational use of disposable and non-disposable equipment.

### BENIGN STRICTURES

The main cause is the failure to identify the structures of the Triangle of Calot. The best treatment is prevention and the most important factor in prevention is beginning the dissection high in the gallbladder infundibulum and dissect in all its circumference the junction of the cystic duct and infundibulum of the gallbladder. Factors reported at this meeting that carry poor prognosis include delayed diagnosis, bile leaks, bile peritonitis, high strictures, multiple previous repairs and cirrhosis. A sutured hepaticojejunostomy was reported in several studies to achieve good results in 76% of patients with an additional 10% of patients improved by reoperation or instrumentation with an ultimate success rate approaching 90% . Anastomosis to viable tissue, lack of tension and adequate dissection of the hilar plate appear to be important factors for success. Stents are not necessary. In the patients at high risk of failure (primarily high strictures above the bifurcation),the use of metal rings or an antecolic jejunal loop or a loop of jejunum attached to the abdominal wall were reported to facilitate percutaneous instrumentation in this difficult subset of patients. A study from the Netherlands confirms the knowledege that mild to moderate elevation of the liver function studies (primarily alkaline phosphatase) following biliary enteric anastomosis is a common occurrence. Clinical cholangitis did not appear to relate to the postoperative liver function studies,ultrasound or CT findings. Therefore,minor and moderate elevations of the alkaline phosphatase do not need to be studied in the asymptomatic patient.

Extrahepatic bile duct injuries secondary to blunt or penetrating trauma are rare and were reported in 0.3% of cases admitted for trauma. The treatment has to be related to the hemodynamic and metabolic stability of the patient and to the associated injuries. Unstable patients require subhepatic drainage,control of bleeding and management of associated injuries and delayed repair. Stable patients with few other injuries may be suitable for initial repair. A study dealing with strictures secondary to pancreatitis confirms the fact that most of these patients require a biliary enteric anastomosis and are not suitable for endoscopic stenting.

We need to decrease the incidence of benign biliary strictures. If they occur, we should avoid compounding the problem. A multi-specialty approach in which the members of the team understand the natural history of the disease and the limitations and advantages of the different therapeutic options is needed. We have seen an increasing number of patients treated with permanent metal expandable endoprothesis or prolonged repeated attempts at dilatation presenting to us with multiple late complications. We should not forget that the median age of patients with bile duct injuries is 40 years of age and therefore, we are interested in the best quality of life and best long-term result at the lower cost in a population with a long life expectancy; often this means a well performed surgical procedure.

### OBSTRUCTIVE JAUNDICE

Several experimental studies confirm the deleterious effects of biliary obstruction in both cellular and humoral defenses.One study also noted increased bacterial overgrowth in the gut secondary in part, to altered motility and suggested the use of Octreatide. An experimental study from Belfast confirms the improvement of Kupffer cell function within 3 weeks of biliary decompression. A clinical study from Thailand suggests that after surgical drainage, the liver functions recover at a median of 6 weeks and the albumin level at approximately 3 weeks. The clinical merits of the experimental work need to be re-addressed. Previous clinical studies on preoperative decompression are not current, frequently used percutaneous techniques and decompression were often used for a short time. Retrospective and nonrandomized studies presented here failed to show benefits of preoperative drainage.

A study using a 20 cm jejunal loop between the hepatic duct and duodenum to allow for long-term instrumentation of high strictures by endoscopic means had a very high failure rate in allowing visualization and instrumentation of the anastomosis. This study contrasts with the use of percutaneous radiologic techniques through an antecolic or subcutaneous loop.

Papers dealing with unusual causes of biliary obstruction were presented. Limited local excision of a cystadenoma or cystadenocarcinoma of the biliary tree appears to be associated to a recurrence rate of 50%, therefore wide resections of the biliary tree with biliary enteric anastomosis are advocated. The management of cholecystobiliary fistula secondary to gallstone disease, the so called Mirizzi syndrome was reviewed. The treatment advocated was dependent on the size and diameter of the fistula and the anatomy of the biliary tree. Treatment varied between repair over a T-tube to a high hepaticojejunostomy. A study from Hamburg confirms that retrograde endoscopy and angiography are the preferred modalities for diagnosing hemobilia. The initial treatment should be embolization. Surgery should be used for failures or for the definitive treatment of underlying processes and includes procedures such as arterial ligation and liver resection. The role of endoscopic and surgical palliation in the management of distal malignant obstruction remains unsettled. A recent report from the Netherlands suggests an advantage for surgical bypass in patients with a life expectancy of more than 6 months and an advantage for endoscopic drainage in patients with shorter survivals. Previous prospective randomized trials comparing endoscopic and surgical palliation have reported surgical mortalities of about 20%, rates currently unacceptable that should be in the 2% range. Periodic analysis of benefits will be required to evaluate the introduction of better stents. At a time of cost containment,one of the many challenges that remains for us in the management of obstructive jaundice, is the design of an algorithm for the diagnosis and management of these patients. This requires an understanding of the natural history of the underlying diseases and the advantages and disadvantages of different diagnostic and therapeutic modalities. Frequently patients with obstructive jaundice are studied indiscriminately with multiple studies, many of which have little relevance in the treatment of that particular patient.

The use of laparoscopic cholecystojejunostomy and gastrojejunostomy for malignant obstructive jaundice in patients who are felt to be unresectable and to have either gastric outlet obstruction or in whom an endoscopic stent is not possible, has been reported and deserves consideration.

### BILIARY MALIGNANCIES

Gallbladder carcinoma has epidemic proportions in some countries. Most patients continue to be diagnosed incidentally at the time of cholecystectomy or late in the course of the carcinoma. Papers presented confirm that the prognosis is primarily related to the depth of invasion of the gallbladder wall and the presence of nodal and metastatic disease. The possibility that in addition to gallstone disease, some of these patients have proliferative changes in the gallbladder mucosa secondary to an abnormal pancreaticobiliary connection was suggested. Patients who are found to have an incidental mucosal or submucosal carcinoma in the cholecystectomy specimen,probably do not require additional treatment. Implantation of tumor in trocar sites and carcinomatosis after laparoscopic cholecystectomy are being increasingly reported.Therefore, if gallbladder cancer is suspected,open cholecystectomy should be done. The management of other stages of the disease remains controversial. In patients with invasion of the subserosa, a wedge resection of the liver and lymphadenectomy has been advocated. Resectability of advanced stages of the disease including the use of hepatic resections and pancreaticoduodenectomies have been reported; however the morbidity and mortality are significant and the impact on survival and quality of life remains unclear. Similarly, the benefits of chemotherapy and radiotherapy are controversial.

Several authors reported their experience with bile duct carcinoma. Better preoperative staging of the disease (ultrasound, CT, angiography, cholangioscopy etc.), the decreased operative mortality for liver surgery and the limited results of non-surgical intubation techniques explains the increasing interest in surgical resection for these tumors. Resectabilities of 30–50% were reported with operative mortalities under 10% and 5 year survivals of up to 30%. The main prognostic factor appears to be the presence of a negative margin. Other factors of poor prognosis include vascular invasion and nodal involvement. Operations are tailored to the extent of the disease and vary from skeletization resection, to different forms of hepatic resection. Emphasis has been made on the frequent involvement of the caudal lobe and the need to resect it. The group at John Hopkins studied the patterns of recurrence in perihilar cholangiocarcinoma confirming that the majority of patients who recur, do so locally. They also note that radiation therapy had no impact on the course of the disease. Resection appears to be associated with the best palliation in those patients who died of the disease. Stenting with expandable stents, although more expensive at the time of placement,appears to be associated to a better long term patency,lower incidence of cholangitis and lower need for re-admissions and stent change than plastic stents. The role of liver transplantation in biliary carcinoma remains an issue of debate. The group at Hanover reported that all their patients with intrahepatic cholangiocellular carcinoma recurred within one year and therefore should not be considered for transplantation. On the other hand, patients transplanted for Klatskin tumors had a 59% one year survival and a 17% five year survival. This survival was correlated to the stage of the disease, primarily nodal status. A study from Nagoya,Japan suggests that in patients with an infected segment or lobe of the liver, the operative mortality and morbidity can be improved by preoperative percutaneous drainage.

### CONGENITAL ANOMALIES

Theories with regard to the pathogenesis of choledocal cysts include: first, an unequal epithelial proliferation during embryogenesis; second, an abnormal common duct pancreatic junction resulting in pancreatic reflux, destruction of the biliary epithelium and changes of the common duct wall. A study presented at this meeting relates the pathogenesis of the disease to the lack of ganglion cells. A study from South Africa on choledocal cysts in adults confirms the high incidence of synchronous or metachronus carcinomas and emphasizes the need for resection.

The peripapillary syndrome, a syndrome generally related to a duodenal diverticulum with biliary obstruction and motility dysfunction of the sphincter is addressed in a paper from China. Transection of the first portion of the duodenum with a Roux-en-y end to side duodenojejunostomy and an end to side hepaticoduodenostomy was associated with good results.

As we approach the closing moments of this Congress, two issues come to mind; first our limited knowledge and second, our increasing need for Gastroenterologists, Endoscopists, Surgeons, Invasive Radiologists, Radiotherapists, Oncologists and others to work as a team in order to better understand the underlying diseases and define the best diagnostic steps and therapies for each particular patient. I believe this Society has this potential. I wish to congratulate the leadership of the IBA and HPB for having united their efforts.

